# Correlation between Pregnancy Outcome and Placental Pathology in COVID-19 Pregnant Women

**DOI:** 10.1155/2022/8061112

**Published:** 2022-08-21

**Authors:** Sara A. Al-Rawaf, Enas T. Mousa, Noora M. Kareem

**Affiliations:** ^1^Department of Gynecology and Obstetrics, Al-Nahrain College of Medicine, Al-Nahrain University, Baghdad, Iraq; ^2^Department of Pathology and Forensic Medicine Department, Al-Nahrain College of Medicine, Al-Nahrain University, Baghdad, Iraq

## Abstract

**Background:**

Vertical transmission of several viruses during pregnancy has been shown to cause adverse fetal outcomes. The question about the possibility of a similar outcome in association with SARS-CoV-2 has been raised in recently published articles. Indeed, the rate of transmission through the placenta to the fetus reported in women with COVID-19 has been shown to form a minority. The aim of this study was to explore the possible histopathological changes in the placenta of pregnant women with COVID-19 after delivery and those changes in the umbilical cord.

**Methods:**

A case-control study including a total of 50 full-term pregnant women with COVID-19 and 60 control pregnant females. Histopathological evaluation of placental tissues and umbilical cords were reported.

**Results:**

The main findings in the umbilical cord were increased thickness of vessels, thrombus formation, endothelins, and narrow lumen; except for the increased thickness of blood vessels, these findings were more frequently seen in women with COVID-19, in comparison with control women in a significant manner (*p* < 0.05). Increased thickness of blood vessels was more significantly observed in the control group compared to the COVID-19 group (*p* < 0.01). Findings of the placenta included avascular villi, fibrin, thrombosis, and meconium macrophage in various combinations. Except for fibrin as the sole findings, all other findings including combinations were more frequently encountered in the study group in comparison to the control group (*p* < 0.05).

**Conclusion:**

Pregnant women with COVID-19 have significant pathological alterations in the placenta and umbilical cord. These findings reflect the capability of SARS-CoV-2 in causing immunological reactions to the placenta, either directly or indirectly, and these pathologies may be linked to the higher rate of adverse neonatal outcomes and maternal admission to the intensive care unit.

## 1. Introduction

Following the initial reports about COVID-19 cases attributed to SARS-CoV-2 in China in late 2019, the virus rapidly spread all over the globe, and reports from various regions of the world confirmed its pandemic nature [1, 2]. By late 2021, more than 200 million subjects were suffering from the viral infection, and more than four million cases of mortality were reported globally [1]. By that time, and in the US alone, the reported cases of mortality exceeded 600,000 patients [1]. It has been suggested that the virus may have an impact on pregnant women, as previous strains of the same virus family including SARS-CoV and MERS have been reported to adversely affect pregnancy and fetal outcomes [3–5]; however, no evidence-based conclusion was available [1]. Vertical transmission of several viruses during pregnancy has been shown to cause adverse fetal outcomes, and the question about the possibility of similar outcomes in association with SARS-CoV-2 has been raised in recently published articles [6]. Indeed, the rate of transmission through the placenta to the fetus reported in women with COVID-19 has been shown to form a minority [7]. The rarity of maternofetal transmission may be attributable to several factors. The virus must first reach the placenta and cross it [1], and the SARS-CoV-2 is known to have a very low level of viremia [8]. Furthermore, the level of receptor expression that aid in facilitating the entry of the virus is very low in placental tissues [9]; however, much controversy exists in the published articles regarding such levels [10]. It has been shown that most of the cases of neonatal SARS-CoV-2 are due to postnatal infection; nevertheless, it has been shown that breastfeeding is safe because of increasing evidence that breast milk is often negative to PCR examination [11], despite some reported positive cases [12]. Although it has been shown that SARS-CoV-2 transmission through the placenta can occur on very rare occasions, there is mounting evidence that SARS-CoV-2 infection in pregnancy is linked to a variety of negative outcomes in pregnancy. A comprehensive review and meta-analysis of generally high-quality studies for the suitable comparison groups indicated that pregnant women infected with SARS-CoV-2 had a higher risk of preeclampsia, preterm birth, and stillbirth than those who did not have SARS-CoV-2 infection [13]. The present study was aiming at exploring the possible histopathological changes of the placenta in pregnant women with COVID-19 after delivery and for those changes in the umbilical cord.

## 2. Methods

This is case-control study that carried out at the Obstetric Department of Al Imamain Al-Kadhimain Teaching Hospital, Baghdad, Iraq. A total of 50 pregnant women in their full-term pregnancy have COVID-19 rendering to the clinical appearances and PCR consequences and 60 control pregnant women with no clinical and laboratory evidence of COVID-19 infection. The study took place within the period from January 2021 to December 2021. There is an assessment of clinical structures and the past obstetrical history, the age, and the gestational age. Also, the maternal and fetal consequences were taken. The outcome of the fetuses was taken and assessed depending on APGAR scores, and also, the neonatal rate for admission to the unit of intensive care. The maternal outcome was assessed through the rate for admission to the unit of intensive care and the need for oxygenation therapy. Additionally, the rate for cesarean section has been assessed in the two groups. Placental tissues and umbilical cord tissues were obtained at the time of birth, and these were transferred into a proper container with fixative (10% formalin) and then were transferred to the central laboratory for histopathological evaluation. Routine formalin-fixed paraffin embedding tissue processing was done followed by hematoxylin and eosin stain. The slides were examined under light microscopy by a well-trained pathologist, and changes were evaluated qualitatively (preferably need two qualified (board certified) histopathologists).

### 2.1. Ethical Considerations

Our study was approved by the institution of the ethical committee, and the formal agreement was issued through the health director. Verbal consent was obtained from each respondent before enrolment in this research project. The aim of the study and the procedures were fully explained to the participants. The ethical approval ID number is 123 in 1-3-2021. Statistical analysis was done by SPSS 22; frequency and percentage were used for categorical data, and mean, median, and SD for continuous data. Chi-square was used for assessing the association between variables; Pearson correlation shows the correlation between continuous data. *T*-test was used for evaluation differences between mean and median of continuous variables. A *p* value less or equal to 0.05 is considered significant.

## 3. Results

A comparison of the general characteristics of women with COVID-19 and those of group control is shown in Table 1. No statistically significant difference was observed concerning age, gestational age, and parity in patients as compared with the control group.

A comparison of chord histopathological findings for the group of COVID-19 with the group for control is illustrated in Table 2. The main findings were a significant increase in the thickness of umbilical cord vessels in 4(8.0%) cases of COVID-19 in comparison to 32(52.0%) control cases, due to an increase in wall thickness of umbilical artery vessels. The mean wall thickness of umbilical arteries was 0.41 ± 0.08 mm in COVID cases and (0.39 ± 0.04) mm in control cases, while the mean wall thickness of umbilical veins in COVID-19 cases was 0.29 ± 0.12 mm, in comparison to (0.28 ± 0.21) in control cases. The main increase in the thickness of the wall was found mainly in the middle tunica layer rather than the intimate layer. Thrombus formation was mainly arterial in origin, and 60% of the COVID-19 group have no thrombus and narrowing of the vessel lumen, while 16% have isolated thrombus, and 8.0% have isolated narrowing of a vessel (24%) without causing the increase in the thickening of the vessels as the endothelial nuclei bulge towards the lumen. Narrowing of the luminal diameter of both umbilical artery and vein in the COVID-19 group compared to the control group was observed in this study. The mean of luminal cross-section area for the umbilical vessels (square mm) were: for the umbilical artery (0.16 ± 0.03) in COVID-19, (0.43 ± 1.08) in the control group, while in the umbilical vein, (0.82 ± .35) in COVID-19, (1.36 ± 0.74) in the control group. The luminal cross-sectional area for umbilical vessels in the COVID-19 group is more narrowing than for the control group which may suggest a change in the vessel wall or the presence of thrombus or endometritis. Also, 24 (48%) cases of COVID-19 had endothelins.

Except for increased thickness of blood vessels, these findings were frequent more in women with COVID-19 in comparison with control women in a significant manner (*p* < 0.05). Increased thickness of blood vessels, however, was more frequently encountered in the group of control compared to the group of study (*p* < 0.01).

A comparison of placental histopathological findings in the group for COVID-19 with a group of control is illustrated in Table 3. These findings included avascular villi, fibrin, thrombosis, and meconium macrophage in various combinations. Except for fibrin as the sole findings, all other findings including combinations were significantly more frequently in a study group in comparison to the control group (*p* < 0.05).

The comparison between outcomes for the maternal and the fetal findings in COVID-19 and the control group is illustrated in Table 4. APGAR score means showed no significant variation between study and control groups at one minute and at 5 minutes (*p* > 0.05). However, the fetal rate for admission to the intensive care unit as well as maternal admission to the intensive care unit was more frequently seen in the study group compared with the control group in a significant manner (*p* < 0.05).

## 4. Discussion

This study is aimed at highlighting the possible link between placental involvement by SARS-CoV-2 and fetal and maternal outcomes in a sample of Iraqi pregnant women. In the current study, we tried to choose a control group with a comparable range of age, range of gestational age, and range of parity to avoid bias in the results attributed to significant variation in these variables. We observed significant variation in the histopathological changes both in the placenta and in the cord. The main changes in the cord were increased thickness of vessels resulting in narrowing of the lumen, thrombus formation, and endothelins. In the placenta, these findings included avascular villi, fibrin, thrombosis, and meconium macrophage in various combinations [14]. In our study, the rates of admission to intensive care unit for both neonates and mothers were more frequently encountered in women with COVID-19 in comparison with pregnant women without COVID-19. We believe that this high ratio for admission to the unit of intensive care is linked with changes seen in the placenta caused by SARS-CoV-2.

COVID-19 promotes more severe disease during pregnancy, according to research [15]. Although a lot of early research lacked a proper comparison group, later, the studies started to compare pregnant and nonpregnant women, with age and comorbidities taken into account (1). The CDC's COVID-19 monitoring system, which includes almost 400,000 people of reproductive age who have symptomatic COVID-19 and were adjusted according to age, race, ethnicity, and medical disorder, yielded some of the most useful data. Women who are pregnant can be hospitalized in an intensive care unit 3 times more than nonpregnant women, 2.9 times require invasive ventilation, 2.4 times require extracorporeal membrane oxygenation, and 1.7 times die than nonpregnant women [16]. These data support our finding that more severe disease is encountered in pregnant ladies, and a significant proportion may be admitted to the intensive care unit and that neonates may require intensive care unit admission more often than the rate seen in healthy pregnancies.

In one previous study, placental tissues from women with COVID-19 were examined and the following features were identified on histological examination: microvascular thromboses were found in decidua and the villous vessels, a scanty lymphocytic inflammation that involve both the decidua and the villi in the placenta basalis with initial agglutination of villous and chronic villitis, intervillous hematoma, and thrombo-hemorrhagic alterations which occur in the vessels of decidual layer, villous infarction, loss of trophoblast, and focal thrombi. These findings were statistically more frequent when compared to placentas of normal women [17]. Other previous reports from placental tissues of women with COVID-19 described no specific histopathological findings [18, 19]. However, significantly, other authors [20] have reported more prominent pathological alterations. Hypercoiling of the umbilical cord, phagocytosis of meconium, and diffuse villous edema were the main changes seen in umbilical cords of babies who were born to women who have COVID-19 [21–30]. These observations support our findings. The higher rate of admission to neonatal intensive care units and maternal intensive care units is suggested to be linked to changes seen in histopathological examination of placental tissues and umbilical cord indicating more severe disease and precipitation to maternal and fetal adverse outcomes.

## 5. Conclusion

Pregnant women with COVID-19 have significant pathological alterations in the placenta and umbilical cord, these findings reflect the ability of SARS-CoV-2 to cause immunological reactions in the placenta, either directly or indirectly, and these pathologies may be linked to the higher rate of adverse neonatal outcomes and maternal admissions to the intensive care unit.

## Figures and Tables

**Table 1:** Comparison of general characteristics of women with COVID-19 and control group.

Characteristic	COVIID-19 *n* = 50	Control group *n* = 60	*p*
Age (years)			
The mean ± SD	26.01 ± 3.19	25.08 ± 2.07	0.231 I NS
The range	18-35	18-35	
Gestational age (weeks)			
The mean ± SD	36.78 ± 1.09	36.29 ± 1.23	0.359 I NS
The range	32-40	35-39	
Parity			
The median (IQR)	3 (2)	3 (2)	0.308 M NS
The range	1 -6	1 -5	

*n*: number of cases; SD: standard deviation; IQR: interquartile range; I: independent samples *t*-test; M: Mann–Whitney *U* test.

**Table 2:** Comparison of umbilical cord histopmorphological findings between COVID-19 group and control group.

Chord histopathological findings	COVID-19 *n* = 50	Control *n* = 60	*p*
Increase thickness of the vessels	4 (8.0%)	32 (53.3%)	<0.001 C^∗∗^
Thrombus	8 (16.0%)	0 (0.0%)	0.001 F^∗∗^
Endotheliitis and narrow lumen	24 (48.0%)	0 (0.0%)	<0.001 C^∗∗^
Narrow lumen and thrombus	20 (40.0%)	0 (0.0%)	<0.001 C^∗∗^
Narrow lumen	4 (8.0%)	0 (0.0%)	0.040 F^∗^
Hypercoiling of umbilical cord	3 (6.0%)	0 (0.0%)	

*n*: number of cases; C: chi-square test; F: Fischer exact test; ^∗^: significant at *p* ≤ 0.05; ^∗∗^: significant at *p* ≤ 0.01.

**Table 3:** Comparison of placental histopathological findings between COVID-19 group and control group.

Placental histopathological findings	COVID-19 *n* = 50	Control *n* = 60	*p*
Avascular villi, fibrin, and thrombosis	4 (8.0%)	0 (0.0%)	0.040 F^∗^
Avascular villi	32 (64.0%)	0 (0.0%)	<0.001 C^∗∗^
Fibrin, thrombosis, and meconium macrophage	12 (24.0%)	0 (0.0%)	<0.001 C^∗∗^
Fibrin and microvascular thrombosis	24 (48.0%)	8 (13.3%)	<0.001 C ^∗∗^
Avascular villi and thrombosis	8 (16.0%)	0 (0.0%)	0.001 F^∗∗^
Thrombosis and meconium macrophage	8 (16.0%)	0 (0.0%)	0.001 F^∗∗^
Avascular villi and meconium macrophage	8 (16.0%)	0 (0.0%)	0.001 F^∗∗^

*n*: number of cases; C: chi-square test; F: Fischer exact test; ^∗^: significant at *p* ≤ 0.05; ^∗∗^: significant at *p* ≤ 0.01; NS: not significant at *p* > 0.05.

**Table 4:** Maternal and fetal outcome contrasted between COVID-19 and control group.

Characteristic	COVID-19 *n* = 50	Control group *n* = 60	*p*
APGAR 1 min			
The mean ± SD	7.31 ± 0.80	7.10 ± 1.03	0.901 I NS
The range	6 -8	6 -8	
APGAR 5 min			
The mean ± SD	7.45 ± 0.71	7.86 ± 0.62	0.411 I NS
The range	6 -8	6 -9	
Admission to NICU			
*n* (%)	9 (18.0%)	3 (5.0%)	0.029 Y^∗^
Maternal outcome (ICU)			
*n* (%)	6 (12.0%)	0 (0.0%)	0.007 F^∗∗^

*n*: number of cases; APGAR: Appearance, Pulse, Grimace, Activity, Respiration; SD: standard deviation; NICU: neonatal intensive care unit; I: independent samples *t*-test; Y: Yates correction test; F: Fischer exact test; NS: not significant at *p* > 0.05; ICU: intensive care unit; ^∗^: significant at *p* ≤ 0.05; ^∗∗^: significant at *p* ≤ 0.01.

## Data Availability

The data used to support the findings of this study are included in the article.
